# Phosphorus Efficiency Mechanisms of Two Wheat Cultivars as Affected by a Range of Phosphorus Levels in the Field

**DOI:** 10.3389/fpls.2018.01614

**Published:** 2018-11-06

**Authors:** Yan Deng, Wan Teng, Yi-Ping Tong, Xin-Ping Chen, Chun-Qin Zou

**Affiliations:** ^1^Key Laboratory of Plant-Soil Interactions, Ministry of Education, Center for Resources, Environment and Food Security, China Agricultural University, Beijing, China; ^2^Hainan Key Laboratory for Sustainable Utilization of Tropical Bioresources, Hainan University, Haikou, China; ^3^The State Key Laboratory of Plant Cell and Chromosome Engineering, Institute of Genetics and Developmental Biology, Chinese Academy of Sciences, Beijing, China; ^4^College of Resources and Environment, Southwest University, Chongqing, China

**Keywords:** root morphology, arbuscular mycorrhizal fungal colonization, acid phosphatase, *TaIPS1.1*, phosphate transporter, phosphorus acquisition efficiency, phosphorus utilization efficiency

## Abstract

Phosphorus (P) efficiency includes both P acquisition efficiency (PAE) and internal P utilization efficiency (PUE). Despite substantial research, genotypic variation in PAE and PUE remains incompletely understood in the field. A 2-year field study was conducted to compare PAE and PUE and related morphological, physiological, and molecular root traits of two winter wheat cultivars (*Triticum aestivum* L. cv. SJZ8 and KN92) in response to six P application rates in a P-deficient calcareous soil. Both cultivars showed similar growth and yield potential at each P supply level, reaching optimal growth at the same P application rate of about 100 kg P ha^-1^. However, the two cultivars differed in how they achieved yield and P efficiency. As P supply increased for both cultivars, root dry weight (RDW), root length density, and expression of the phosphate transporter gene *TaPHT1.2* in roots initially increased and then stabilized, but arbuscular mycorrhizal fungal colonization, rhizosphere acid phosphatase activity, expressions of the P-starvation marker gene *TaIPS1.1* and the purple acid phosphatase gene *TaPAP16* in roots initially decreased and then stabilized. To enhance P acquisition when the P supply was deficient, KN92 modified the morphology of its roots, while SJZ8 increased the physiological activities in its roots. With an adequate P supply, high expression of *TaPHT1.2* in roots might account for efficient P uptake for both cultivars, especially for KN92. Although P uptake per RDW was similar for both cultivars at anthesis, PAE was higher for KN92 than SJZ8 in terms of total P uptake in aboveground parts, whereas shoot and grain PUE were higher in SJZ8 than in KN92, mainly during the reproductive growth stage. These results indicate that P efficiency is under genotypic control at all P supply levels tested in both wheat cultivars, and that the two cultivars depend on different root strategies for P acquisition and utilization in response to changes in the P supply.

## Introduction

Phosphorus (P) fertilizers have been widely applied in many intensive cropping systems (Lott et al., 2011), but crops can usually use only 10–30% of the fertilizer P in the application year (Syers et al., 2008). Enhancing P efficiency has long been a key challenge in intensive cropping systems (Shenoy and Kalagudi, 2005; Schröder et al., 2011). Because most fertilizer P remains in the soil, Sattari et al. (2012) estimated that residual soil P from intensive fertilization in the past would contribute significantly to future crop production, with a considerable lag time. Thus, an increased plant capacity to efficiently use the residual P will be very helpful in crop production. The decreasing availability of rock phosphate as a source of P fertilizer and the increasing awareness of the negative environmental consequences of high P fertilizer input have also increased the interest in enhancing the efficiency of P acquisition and utilization by plants (Li et al., 2011; Richardson et al., 2011; Tian et al., 2012; Clemens et al., 2016).

P efficiency can be enhanced by improving P scavenging and uptake (P acquisition efficiency, PAE) and, more economically, by improving P utilization in the plant (P utilization efficiency, PUE) (Wang et al., 2010; Rose and Wissuwa, 2012; Veneklaas et al., 2012; Clemens et al., 2016). To optimize access to soil P when P is limiting, plants have evolved highly specialized adaptive mechanisms, including morphological, physiological, and molecular modifications, e.g., an increased root/shoot ratio, an increase in the number of root hairs, association with arbuscular mycorrhizal fungi (AMF), synthesis and release of phosphatases and organic acids, and enhanced expression of phosphate transporters (Richardson et al., 2009, 2011; Péret et al., 2011). Many studies have shown that substantial genotypic variations exist in these traits, making it possible to breed P-efficient crop cultivars with higher PAE (Gahoonia et al., 1997; Gahoonia and Nielsen, 2004; Lynch, 2007). However, the beneficial traits mentioned above are often depressed by increased P supply (Ma et al., 2001; Shen et al., 2002; Nagy et al., 2006; Covacevich et al., 2007; Teng et al., 2013). In many intensive cropping systems, soil P content has exceeded crop needs because of excessive P fertilizer application (Sattari et al., 2012). Developing crop cultivars whose PAE mechanisms are not depressed under high-P conditions would be useful because such crop plants could use more residual soil P and thereby require less input of P fertilizer. As stated by Lynch (2007), an ideal P-efficient crop genotype with traits related to P acquisition should have relatively high yield potential in infertile soils of low-input agroecosystems, and should require reduced P fertilizer in high-input agroecosystems.

In their review, Wang et al. (2010) indicated that PAE could be more important than PUE when the P supply is limited, while PUE could be more important when the P supply is adequate. In addition, a crop’s reliance on PAE or PUE is thought to be mostly under genotypic control and differ among crops species and genotypes within the same species (Wang et al., 2010). In spring wheat, for example, P efficiency was mostly determined by PAE under P-deficient conditions but by PUE under P-sufficient conditions (Manske et al., 2001). In maize, however, it seems that PAE rather than PUE determines P efficiency under both low and high P conditions, at least in acidic soils (Parentoni and Souza Júnior, 2008). Because root system modifications require additional carbon input, crops with better root traits for efficient P uptake might have to sacrifice carbohydrates needed for higher yield (Manske and Vlek, 2002; Lynch and Ho, 2005). Researchers have therefore speculated that, in intensive cropping systems, crop genotypes with high PUE rather than high PAE may be more promising for increasing P efficiency (Wang et al., 2010).

In China, wheat usually consumes higher P input per unit area from fertilizer and other sources but has a lower P efficiency than maize or rice (Ma et al., 2011). Improving P efficiency to reduce P fertilizer input is therefore required for sustainable P use in wheat production in China. Considerable research has been directed at selecting and breeding P-efficient wheat genotypes (Davies et al., 2002; Wang et al., 2005), and genotypic variations in PAE and PUE for wheat have been widely reported (Batten, 1992; Horst et al., 1993; Manske et al., 2001; Wang et al., 2005; Gunes et al., 2006). Most of these studies, however, were conducted under controlled greenhouse conditions, and most compared PAE and PUE for different wheat genotypes under low and adequate P conditions but failed to consider sub-adequate (with P levels between low and adequate levels) and excessive P conditions. Because the expression of P efficiency by wheat genotypes might differ under greenhouse and field conditions (Gunes et al., 2006), field-based studies with low, sub-adequate, adequate, and excessive P supplies are needed. Thus the objectives of this study were 1) to compare the PAE and PUE of two wheat cultivars as affected by a wide range of P supply in the field, and 2) to determine the mechanisms underlying the observed differences in PAE and PUE. The second objective involved the characterization of the morphological, physiological, and molecular responses of the roots of both cultivars to the wide range of P supply in the field.

## Materials and Methods

### Wheat Cultivars

Winter wheat (*Triticum aestivum* L.) cultivars Shijiazhuang8 (abbreviated SJZ8) and Kenong9204 (abbreviated KN92) were used in the field experiment. Commercially released in 2003, both cultivars are adapted to the North China Plain, which is the main winter wheat production area in China. Under suitable growth conditions, SJZ8 has a plant height about 75 cm, produces 32 grains per ear and weighs 45 g per 1000 grains; while KN92 has a plant height of 74 cm, produces 35 grains per ear and weighs 40 g per 1000 grains.

### Field Site Description

The experiment was conducted at the Quzhou Experimental Station of China Agricultural University, Quzhou County, Hebei Province, China (36°52′N, 115°02′E). Influenced by a warm–temperate continental monsoon climate, this area has an annual mean temperature of 13.2°C and an annual mean precipitation of 494 mm. Only about 30% of the precipitation falls during the winter wheat season. The soil is calcareous and has an alluvial loam texture. Soil basic properties in the 0–30 cm layer were as follows: pH 7.3 (water : soil = 2 : 1), organic matter 10.3 g kg^-1^, total N 0.67 g kg^-1^, available P (Olsen-P) 7.0 mg kg^-1^, and exchangeable K 74 mg kg^-1^.

### Experimental Design

One trial of the experiment was conducted in the 2009–2010 winter wheat growing season (recorded as 2010), and a second trial was conducted in the 2010–2011 winter wheat growing season (recorded as 2011). In each trial, wheat was sown in early October and harvested in mid-June of the following year. To rapidly obtain a range of soil P supply from deficiency to excess, P was applied in each trial at six application rates: 0, 25, 50, 100, 200, and 400 kg P ha^-1^ (Soil Olsen-P status at harvest was 5.7–59.7 mg kg^-1^ in 2010 season, and 4.7–55.5 mg kg^-1^ in 2011 season, respectively). Each application rate (treatment) in each trial (year) was represented by four replicate plots; each plot was 43.2 m^2^ (5.4 m × 8 m). P was always applied in the form of calcium superphosphate before seed sowing, when it was mixed into the topsoil, together with 75 kg N ha^-1^ as urea and 50 kg K ha^-1^ as potassium sulfate, by plowing. Another 150 kg N ha^-1^ as urea was top-dressed at vegetation stage for all plots. In each season, both cultivars were sown at a similar seedling density, with a row spacing of 20 cm in 2010 and 15 cm in 2011. Irrigation and pesticides (e.g., lambda-cyhalothrin, imidacloprid, avermectin, and tebuconazole) were applied as needed according to conventional practice to ensure the absence of water deficiency and pest and disease damage.

### Sampling and Measurements

At vegetation, anthesis, and maturity stages in 2010 and 2011, aboveground parts of the wheat plants were randomly sampled in two adjacent rows (0.5 m per row) per plot. Plant samples were oven-dried to determine aboveground plant dry weight. P concentrations of shoots, straw, and grain were then measured using the vanado-molybdate method (Shi, 1986). Aboveground total P uptake (TPU) at each sampling time was calculated based on plant dry weight and the P concentration in the tissues. Grain yield was determined at maturity by harvesting wheat plants in a 6-m^2^ area in the center of each plot that had not been previously sampled.

After aboveground parts were cut, root samples from the 0–20 cm soil layer were collected in each plot at anthesis in 2010 and 2011 to measure root AMF colonization. Fresh roots were washed and cut into 1-cm segments. Subsamples of 0.5 g were then randomly taken, cleared in 10% (w/v) KOH, and stained with Trypan blue in a 90°C water bath (Feng et al., 2003). AMF colonization of roots was assessed by examining 30 randomly selected stained root segments with a light microscope according to Trouvelot et al. (1986).

Root samples for measurement of root morphological traits were also taken at anthesis in both years. Root samples were collected in whole treatments in 2010 but were collected only from plots treated with 0, 50, 100, and 400 kg P ha^-1^ in 2011. To sample roots, soil block which grew two rows of plants was collected from each plot. In 2010, each soil block extended 40 cm perpendicular to the row, 20 cm parallel to the row, and 60 cm deep. In 2011, each soil block extended 30 cm perpendicular to the row, 10 cm parallel to the row, and 60 cm deep. All visible wheat roots in each soil block were manually removed and washed. To determine root length density (RLD, root length per unit soil volume), root samples were first immersed in water in a transparent plastic tray (30 cm long × 20 cm wide × 3 cm high) and imaged with a scanner (Epson Expression 1600, Seiko Epson, Nagano, Japan). These images were then analyzed with WinRhizo software (Regent Instrument Inc., Quebec, QC, Canada) to determine total root length. RLD in the 0–60 cm soil layer was calculated based on the total root length and the volume of the block. After roots were scanned, root dry weight (RDW) in the 0–60 cm soil layer was determined based on the weight of the oven-dried samples and soil volume per ha.

Rhizosphere acid phosphatase (APase) activity was measured at vegetation stage in 2010 and at anthesis stages in 2011, respectively. In each year, five plants per plot were randomly chosen and carefully dug out with roots. These plants were gently shaken to remove soil loosely adhering to the roots; the soil that tightly adhered to the roots was considered rhizosphere soil. Roots with adhering rhizosphere soil were dipped into 50 mL of a 0.2 mM CaCl_2_ solution and shaken gently for 1 min to remove rhizosphere soil (Pearse et al., 2006). A sub-sample (5 ml) of the rhizosphere soil suspension was stored at -20°C prior to analysis. APase activity in the rhizosphere was determined with a spectrophotometer (UVmini-1240, Shimadzu, Japan) by measuring absorptance of *p*-nitrophenol at 405 nm as described by Alvey et al. (2001).

Root samples for gene expression analysis were taken at anthesis in 2011 (but not in 2010) in all the plots. Roots of five randomly selected plants were carefully collected, quickly washed, and stored in liquid N_2_ until analyzed. Expression levels of *TaIPS1.1* (P-starvation marker gene) (Teng et al., 2013), *TaPAP16* (purple APase gene), and *TaPHT1.2* (phosphate transporter gene) (Davies et al., 2002) were analyzed. Total RNA extraction, cDNA synthesis, real-time quantitative reverse transcription PCR (qRT-PCR), and calculation of transcription levels were performed as described by Teng et al. (2013).

P acquisition efficiency was determined in two ways. In one case, PAE was equivalent to TPU per ha at the indicated growth stage. To compare the capacity of roots to absorb P from soil, however, PAE was also expressed as the P absorption efficiency of roots (RPAE), i.e., as the quantity of P accumulated in the aboveground plant material per unit of RDW (Pan et al., 2008). Because most P uptake occurs by the time of anthesis in wheat (Masoni et al., 2007), RDW in the 0–60 cm soil layer at anthesis was used to calculate RPAE. As described by Rose and Wissuwa (2012), PUE at the vegetative stage (termed shoot PUE) was calculated as shoot dry weight per unit P in shoots, while PUE at maturity (termed grain PUE) was calculated as the grain yield per unit P accumulated in all aboveground plant materials.

### Statistical Analysis

Data for aboveground plant dry weight, grain yield, tissue P concentration, TPU, RPAE, shoot PUE, and grain PUE were subjected to three-way analysis of variance (ANOVA; year, cultivar and P level with their interactions) using SPSS statistical software (SPSS 13.0, United States). For root parameters, two-way ANOVA (cultivar and P level with their interaction) was firstly applied, then means between P levels were compared by Turkey test (*P* < 0.05) for each cultivar, and means at each P level between two cultivars were compared by paired-samples Student *T*-test (*P* < 0.05). Graphs were generated using SigmaPlot software (SigmaPlot 10.0, United States). All the data results are expressed as mean ± standard error (SE) of four replications.

## Results

### Plant Growth and Plant P Status

Aboveground plant dry weight and grain yield for both cultivars were significantly affected by cropping year, P application level, and their interaction (Table 1). Plant dry weight and grain yield of the two cultivars generally increased considerably as the P application rate increased up to 100 kg P ha^-1^; above this P level, no further significant increments were obtained. At each sampling time and for each P application level, aboveground dry weight did not significantly differ between the two cultivars.

**Table 1:** Aboveground plant dry weight at vegetation, anthesis, and maturity stages, and grain yield for SJZ8 and KN92 in 2010 and 2011.

		Aboveground plant dry weight (t ha^-1^)							
Year	P level								
	(kg ha^-1^)	Vegetation		Anthesis		Maturity		Grain yield (t ha^-1^)	
		SJZ8	KN92	SJZ8	KN92	SJZ8	KN92	SJZ8	KN92
2010	0	0.81 ± 0.15	0.97 ± 0.03	3.29 ± 0.16	3.89 ± 0.38	6.99 ± 0.22	7.62 ± 0.46	3.38 ± 0.17	3.50 ± 0.35
	25	1.20 ± 0.16	1.37 ± 0.06	4.55 ± 0.27	4.60 ± 0.36	8.67 ± 0.30	8.24 ± 0.19	4.37 ± 0.09	4.13 ± 0.14
	50	1.49 ± 0.06	1.40 ± 0.13	5.37 ± 0.47	5.48 ± 0.20	8.81 ± 0.15	9.77 ± 0.22	4.67 ± 0.12	4.42 ± 0.17
	100	1.59 ± 0.11	1.63 ± 0.11	5.84 ± 0.19	6.89 ± 0.56	11.75 ± 0.64	10.90 ± 0.52	5.70 ± 0.24	5.24 ± 0.17
	200	1.85 ± 0.23	1.98 ± 0.17	6.27 ± 0.44	7.21 ± 0.36	11.99 ± 0.88	12.54 ± 0.45	5.61 ± 0.34	5.92 ± 0.40
	400	1.90 ± 0.09	1.92 ± 0.33	6.72 ± 0.22	7.01 ± 0.26	12.57 ± 0.81	12.61 ± 0.30	5.98 ± 0.54	5.66 ± 0.40
2011	0	1.33 ± 0.16	1.31 ± 0.27	3.35 ± 0.41	3.84 ± 0.22	5.51 ± 0.59	6.79 ± 0.31	2.69 ± 0.32	3.43 ± 0.12
	25	1.87 ± 0.11	1.79 ± 0.05	6.70 ± 0.71	7.20 ± 0.38	10.43 ± 0.27	9.35 ± 0.44	5.37 ± 0.14	4.86 ± 0.20
	50	2.80 ± 0.19	2.10 ± 0.28	9.64 ± 0.41	7.88 ± 0.45	12.49 ± 0.76	10.98 ± 0.44	6.31 ± 0.30	5.77 ± 0.33
	100	2.98 ± 0.36	3.06 ± 0.22	10.69 ± 0.99	10.91 ± 1.02	12.25 ± 0.82	11.68 ± 0.62	6.12 ± 0.28	5.89 ± 0.25
	200	3.34 ± 0.19	2.95 ± 0.22	10.16 ± 0.75	10.29 ± 0.95	13.28 ± 0.93	12.23 ± 0.33	6.49 ± 0.39	6.33 ± 0.50
	400	3.40 ± 0.19	2.92 ± 0.01	10.33 ± 0.74	9.64 ± 0.75	13.82 ± 0.69	13.41 ± 0.78	6.42 ± 0.17	6.80 ± 0.32
Year (Y)		^∗∗∗^		^∗∗∗^		^∗^		^∗∗^	
Cultivar (C)		ns		ns		ns		ns	
P level (P)		^∗∗∗^		^∗∗∗^		^∗∗∗^		^∗∗∗^	
Y × C		ns		ns		^∗^		ns	
Y × P		^∗^		^∗∗∗^		^∗∗∗^		^∗∗∗^	
C × P		ns		ns		ns		ns	
Y × C × P		ns		ns		ns		ns	

*Values are means ± SE of four replicates. ns, ^∗^, ^∗∗^, and ^∗∗∗^ indicate no significant difference and significant difference at *P* < 0.05, 0.01, and 0.001 level, respectively*.

For both cultivars, P concentrations in tissues were significantly affected by cropping year and P application level (Table 2). In both years, P concentrations in tissues of both cultivars increased as the P application level increased. Regardless of P level and cropping year, P concentrations in tissues were higher in KN92 than in SJZ8 after the vegetation stage and especially at maturity when straw and grain P concentrations significantly differed between the two cultivars.

**Table 2:** Shoot P concentration (SHPc) at vegetation and anthesis stages, straw P concentration (STPc), and grain P concentration (GPc) at maturity stage for SJZ8 and KN92 in 2010 and 2011.

Year	P level	SHPc-Vegetation		SHPc-Anthesis		STPc-Maturity		GPc-Maturity	
	(kg ha^-1^)	(mg kg^-1^)		(mg kg^-1^)		(mg kg^-1^)		(mg kg^-1^)	
		SJZ8	KN92	SJZ8	KN92	SJZ8	KN92	SJZ8	KN92
2010	0	2.13 ± 0.06	1.91 ± 0.04	2.13 ± 0.09	2.28 ± 0.16	0.29 ± 0.02	0.35 ± 0.04	2.75 ± 0.06	3.29 ± 0.05
	25	2.16 ± 0.05	1.94 ± 0.08	2.18 ± 0.06	2.26 ± 0.05	0.33 ± 0.04	0.40 ± 0.02	2.97 ± 0.09	3.40 ± 0.11
	50	2.20 ± 0.06	2.23 ± 0.22	2.21 ± 0.02	2.31 ± 0.06	0.32 ± 0.04	0.37 ± 0.03	3.04 ± 0.06	3.52 ± 0.14
	100	2.56 ± 0.09	2.74 ± 0.09	2.41 ± 0.03	2.42 ± 0.08	0.34 ± 0.03	0.48 ± 0.04	3.17 ± 0.05	3.56 ± 0.07
	200	2.71 ± 0.17	3.02 ± 0.18	2.44 ± 0.06	2.58 ± 0.08	0.37 ± 0.03	0.49 ± 0.04	3.22 ± 0.07	3.68 ± 0.05
	400	3.46 ± 0.19	3.65 ± 0.09	2.51 ± 0.11	2.80 ± 0.09	0.42 ± 0.02	0.55 ± 0.05	3.35 ± 0.08	3.87 ± 0.10
2011	0	1.51 ± 0.05	1.50 ± 0.12	1.32 ± 0.16	1.46 ± 0.08	0.20 ± 0.02	0.22 ± 0.01	2.25 ± 0.07	2.68 ± 0.14
	25	1.67 ± 0.04	1.62 ± 0.09	1.56 ± 0.05	1.65 ± 0.08	0.20 ± 0.00	0.26 ± 0.02	2.55 ± 0.10	2.97 ± 0.15
	50	1.90 ± 0.14	1.73 ± 0.03	1.72 ± 0.05	1.82 ± 0.12	0.27 ± 0.02	0.30 ± 0.03	2.81 ± 0.19	3.14 ± 0.07
	100	2.49 ± 0.15	2.18 ± 0.13	1.85 ± 0.07	2.04 ± 0.12	0.34 ± 0.02	0.39 ± 0.05	2.79 ± 0.06	3.38 ± 0.11
	200	2.51 ± 0.07	2.80 ± 0.08	1.92 ± 0.06	2.13 ± 0.08	0.35 ± 0.03	0.42 ± 0.01	3.02 ± 0.07	3.45 ± 0.05
	400	3.17 ± 0.19	3.35 ± 0.09	2.18 ± 0.15	2.30 ± 0.14	0.38 ± 0.02	0.46 ± 0.01	3.28 ± 0.10	3.36 ± 0.08
Year (Y)		^∗∗^		^∗∗∗^		^∗∗^		^∗∗∗^	
Cultivar (C)		ns		ns		^∗^		^∗∗∗^	
P level (P)		^∗∗∗^		^∗∗∗^		^∗∗∗^		^∗∗∗^	
Y × C		ns		ns		ns		ns	
Y × P		ns		ns		ns		ns	
C × P		ns		ns		ns		ns	
Y × C × P		ns		ns		ns		ns	

*Values are means ± SE of four replicates. ns, ^∗^, ^∗∗^, and ^∗∗∗^ indicate no significant difference and significant difference at *P* < 0.05, 0.01, and 0.001 level, respectively*.

### P Acquisition Efficiency and Utilization Efficiency

P acquisition efficiency in terms of TPU was significantly influenced by cropping year, P level, and their interaction (Table 3). For both cultivars, TPU increased as the P application level increased at each sampling time in the two cropping years. TPU tended to be greater in KN92 than in SJZ8 at anthesis, and the difference became significant (*P* < 0.01) at maturity (Table 3). On the other hand, RPAE did not significantly differ between the two cultivars at anthesis in each cropping year (Table 4). RPAE was significantly affected by P application level and cropping year. With increasing P application level, RPAE for both cultivars tended to increase. For both cultivars, RPAE was lower in 2011 than in 2010, especially in the absence of P application (0 kg P ha^-1^).

**Table 3:** Aboveground total P uptake (TPU) at vegetation, anthesis, and maturity stages for SJZ8 and KN92 in 2010 and 2011.

Year	P level	TPU-Vegetation		TPU-Anthesis		TPU-Maturity	
	(kg ha^-1^)	(kg ha^-1^)		(kg ha^-1^)		(kg ha^-1^)	
		SJZ8	KN92	SJZ8	KN92	SJZ8	KN92
2010	0	1.75 ± 0.38	1.85 ± 0.04	6.96 ± 0.07	8.96 ± 0.41	10.35 ± 0.56	12.96 ± 0.33
	25	2.69 ± 0.60	2.65 ± 0.19	9.92 ± 0.66	10.36 ± 0.65	14.38 ± 0.61	15.71 ± 0.70
	50	3.28 ± 0.16	3.18 ± 0.54	11.90 ± 0.33	12.62 ± 0.51	15.53 ± 0.46	17.51 ± 0.86
	100	4.09 ± 0.41	4.49 ± 0.42	14.11 ± 0.62	16.53 ± 0.96	20.17 ± 0.57	21.41 ± 0.64
	200	4.93 ± 0.51	5.96 ± 0.53	15.30 ± 0.68	18.70 ± 0.69	20.46 ± 0.71	25.09 ± 0.28
	400	6.51 ± 0.18	6.96 ± 0.47	16.94 ± 0.58	19.68 ± 0.56	22.61 ± 0.59	25.73 ± 0.40
2011	0	2.02 ± 0.28	1.92 ± 0.35	4.28 ± 0.40	5.61 ± 0.39	6.57 ± 0.63	10.00 ± 0.82
	25	3.13 ± 0.19	2.90 ± 0.10	10.43 ± 0.34	11.93 ± 0.97	14.73 ± 0.86	15.63 ± 0.27
	50	5.26 ± 0.57	3.63 ± 0.50	16.62 ± 0.50	14.25 ± 0.84	19.34 ± 0.57	19.68 ± 0.33
	100	7.34 ± 0.33	6.76 ± 0.88	19.88 ± 0.96	22.43 ± 0.73	19.14 ± 0.82	22.16 ± 0.83
	200	7.40 ± 0.50	8.29 ± 0.73	19.43 ± 0.99	21.95 ± 0.60	22.03 ± 0.47	24.28 ± 0.65
	400	10.82 ± 0.76	9.79 ± 0.24	22.45 ± 0.47	22.13 ± 0.64	23.88 ± 0.91	25.93 ± 0.55
Year (Y)		^∗∗∗^		^∗∗^		ns	
Cultivar (C)		ns		ns		^∗∗^	
P level (P)		^∗∗∗^		^∗∗∗^		^∗∗∗^	
Y × C		ns		ns		ns	
Y × P		^∗∗^		^∗∗^		^∗∗∗^	
C × P		ns		ns		ns	
Y × C × P		ns		ns		ns	

*Values are means ± SE of four replicates. ns, ^∗^, ^∗∗^, and ^∗∗∗^ indicate no significant difference and significant difference at *P* < 0.05, 0.01, and 0.001 level, respectively*.

**Table 4:** P acquisition efficiency by roots (RPAE) at anthesis, shoot P utilization efficiency (Shoot PUE) at vegetation and anthesis stages, and grain P utilization efficiency (Grain PUE) at maturity stage for SJZ8 and KN92 in 2010 and 2011.

Year	P level	RPAE-Anthesis		Shoot PUE-Vegetation		Shoot PUE-Anthesis		Grain PUE-Maturity	
	(kg ha^-1^)	(g P kg^-1^ RDW)		(kg SDW kg^-1^ P)		(kg SDW kg^-1^ P)		(kg GY kg^-1^ P)	
		SJZ8	KN92	SJZ8	KN92	SJZ8	KN92	SJZ8	KN92
2010	0	12.1 ± 0.46	13.0 ± 1.20	472 ± 14	524 ± 11	472 ± 20	446 ± 32	327 ± 5	269 ± 4
	25	16.7 ± 0.69	14.9 ± 0.80	479 ± 55	519 ± 21	460 ± 12	442 ± 9	304 ± 9	263 ± 6
	50	18.6 ± 1.10	17.4 ± 0.40	455 ± 13	460 ± 40	452 ± 4	434 ± 12	301 ± 8	253 ± 11
	100	19.5 ± 1.67	20.2 ± 0.71	392 ± 13	366 ± 12	414 ± 5	415 ± 13	284 ± 6	246 ± 7
	200	19.2 ± 1.11	23.5 ± 1.41	374 ± 26	335 ± 18	410 ± 9	388 ± 11	275 ± 6	236 ± 3
	400	20.8 ± 0.61	24.3 ± 0.73	292 ± 18	274 ± 7	401 ± 17	359 ± 12	263 ± 8	220 ± 8
2011	0	6.5 ± 0.26	7.1 ± 0.31	665 ± 21	678 ± 57	790 ± 22	690 ± 37	407 ± 10	347 ± 19
	25	–	–	599 ± 13	622 ± 38	642 ± 18	610 ± 33	367 ± 13	313 ± 16
	50	18.8 ± 1.28	14.4 ± 0.72	536 ± 36	579 ± 9	583 ± 17	556 ± 34	330 ± 22	294 ± 8
	100	17.6 ± 0.93	19.9 ± 1.85	420 ± 51	464 ± 27	543 ± 21	495 ± 31	320 ± 7	266 ± 7
	200	–	–	399 ± 12	358 ± 10	521 ± 15	472 ± 17	296 ± 7	260 ± 5
	400	21.8 ± 0.30	21.4 ± 0.42	319 ± 19	299 ± 8	466 ± 30	440 ± 28	270 ± 10	262 ± 3
Year (Y)		^∗^		^∗∗^		^∗∗∗^		^∗∗∗^	
Cultivar (C)		ns		ns		ns		^∗∗∗^	
P level (P)		^∗∗∗^		^∗∗∗^		^∗∗∗^		^∗∗∗^	
Y × C		ns		ns		ns		ns	
Y × P		ns		^∗^		^∗∗∗^		^∗∗^	
C × P		ns		ns		ns		ns	
Y × C × P		ns		ns		ns		ns	

*Values are means ± SE of four replicates. ns, ^∗^, ^∗∗^, and ^∗∗∗^ indicate no significant difference and significant difference at *P* < 0.05, 0.01, and 0.001 level, respectively*.

At all three sampling stages, PUE, expressed as shoot or grain produced per unit of P uptake in aboveground plant materials, was strongly affected by cropping year, P application level, and their interaction (Table 4). Unlike RPAE, both shoot and grain PUE decreased with increasing P application level for both cultivars in both cropping years. Both cultivars had higher shoot and grain PUE values in 2011 than 2010. Each year at the vegetation stage, SJZ8 and KN92 did not significantly differ in shoot PUE at the same P application level. From the anthesis stage on, shoot PUE was slightly higher for SJZ8 than KN92 at the same P application level, and grain PUE was much higher for SJZ8 than KN92 at harvest.

### Root Morphological, Physiological, and Molecular Responses

**FIGURE 1:**
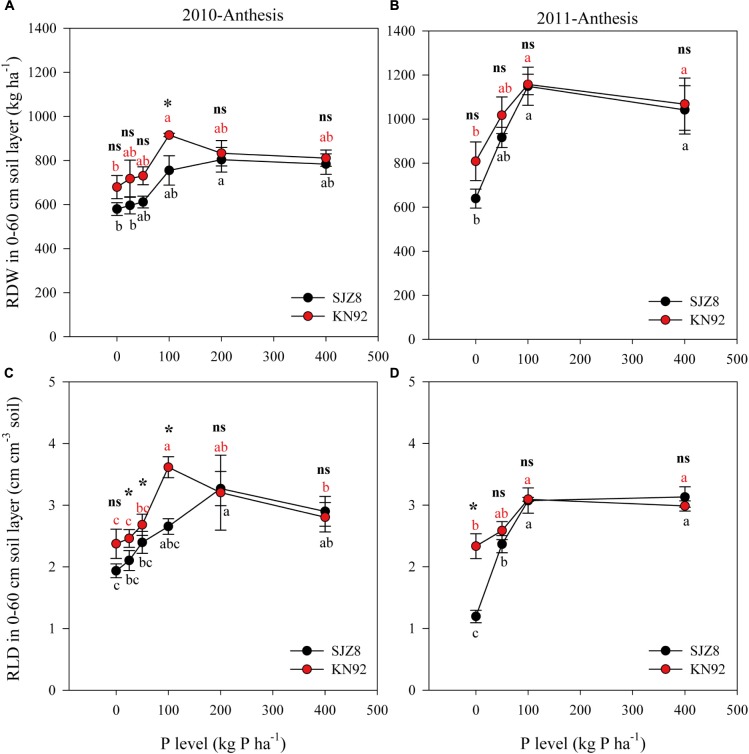
Root dry weight (RDW) **(A,B)** and root length density (RLD) **(C,D)** in the 0–60 cm soil layer for SJZ8 (black) and KN92 (red) at anthesis stage in 2010 and 2011. Values are means ± SE of four replicates. Within each cultivar, different lowercase letters indicate significant difference between P application levels (Turkey test, *P* < 0.05); ns and ^∗^ indicate no significant difference and significant difference, respectively, between SJZ8 and KN92 at the same P application level (Student *T*-test, *P* < 0.05).

Root growth was significantly influenced by P level (Table 5). In 2010, with increasing P application level, RDW at anthesis gradually increased up to the rate of 100 kg P ha^-1^ for KN92 and 200 kg P ha^-1^ for SJZ8; at higher application levels, RDW decreased a little and then remained stable (Figure 1A). Similar trends were obtained in 2011, with RDW reaching the highest value at 100 kg P ha^-1^ for both cultivars (Figure 1B). Responses of RLD to P application level for the two cultivars were similar to the responses of RDW in both years (Figures 1C,D). Significant difference in root growth displayed between two cultivars (Table 5). RDW and especially RLD were higher for KN92 than SJZ8, mainly when the P application level was <100 kg P ha^-1^.

**Table 5:** Two-way analysis of variance of the effects of cultivar and P level with their interaction on tested root parameters at each sampling time for two wheat cultivars grown under varied P application levels in the field. The probabilities of *F*-value are shown.

Root parameters	Source of variation		
	Cultivar	P level	C × P
	(C)	(P)	
RDW (2010-Anthesis)	0.002	<0.001	0.702
RDW (2011-Anthesis)	0.203	<0.001	0.751
RLD (2010-Anthesis)	0.040	<0.001	0.359
RLD (2011-Anthesis)	0.006	<0.001	0.001
AMF colonization (2010-Anthesis)	0.001	<0.001	0.002
AMF colonization (2011-Anthesis)	<0.001	<0.001	<0.001
Rhizosphere APase activity (2010-Vegetation)	0.037	0.003	0.105
Rhizosphere APase activity (2011-Anthesis)	<0.001	<0.001	<0.001
Root *TaIPS1.1* expression (2011-Anthesis)	0.147	<0.001	0.906
Root *TaPAP16* expression (2011-Anthesis)	0.008	<0.001	0.633
Root *TaPHT1.2* expression (2011-Anthesis)	<0.001	<0.001	0.367

*RDW, root dry weight; RLD, root length density; AMF, arbuscular mycorrhizal fungi; APase, acid phosphatase*.

**FIGURE 2:**
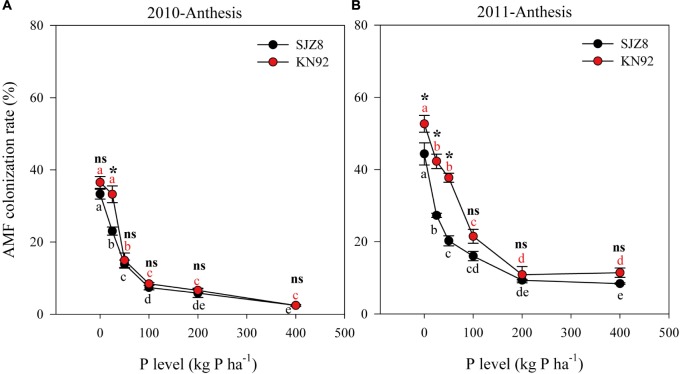
Arbuscular mycorrhizal fungi (AMF) colonization of roots for SJZ8 (black) and KN92 (red) at anthesis stage in 2010 **(A)** and 2011 **(B)**. Values are means ± SE of four replicates. Within each cultivar, different lowercase letters indicate significant difference between P application levels (Turkey test, *P* < 0.05); ns and ^∗^ indicate no significant difference and significant difference, respectively, between SJZ8 and KN92 at the same P application level (Student *T*-test, *P* < 0.05).

In both cropping years, AMF colonization was significantly influenced by cultivar, P level and their interaction (Table 5). Consistently, AMF colonization of both cultivars gradually decreased as the P application level increased up to 200 kg P ha^-1^; above that level, AMF colonization was low and stable (Figure 2). AMF colonization was higher for KN92 than for SJZ8 when the P application level was below the optimum for growth (<100 kg P ha^-1^), especially in 2011 (Figure 2B).

**FIGURE 3:**
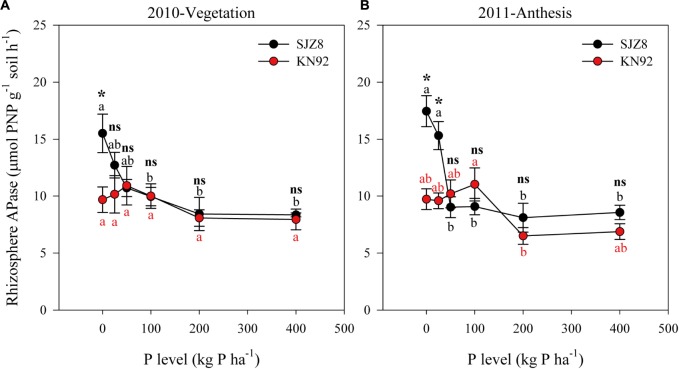
APase activity in rhizosphere soil of SJZ8 (black) and KN92 (red) at vegetation stage in 2010 **(A)** and at anthesis stage in 2011 **(B)**. Values are means ± SE of four replicates. Within each cultivar, different lowercase letters indicate significant difference between P application levels (Turkey test, *P* < 0.05); ns and ^∗^ indicate no significant difference and significant difference, respectively, between SJZ8 and KN92 at the same P application level (Student *T*-test, *P* < 0.05).

The responses of rhizosphere APase activity to P application level differed between the two wheat cultivars (Figure 3 and Table 5). At both sampling times, rhizosphere APase activity for SJZ8 significantly decreased as the P application level increased up to 50 kg P ha^-1^; at higher P application levels, the rhizosphere APase activity did not significantly change for SJZ8. Unlike SJZ8, the rhizosphere APase activity for KN92 tended to increase as the P application level increased from 0 to 50 kg P ha^-1^ in 2010 and from 0 to 100 kg P ha^-1^ in 2011, and then gradually dropped to a stable level with higher P application levels. When the P application level was <50 kg P ha^-1^, rhizosphere APase activity was significantly higher for SJZ8 than KN92.

**FIGURE 4:**
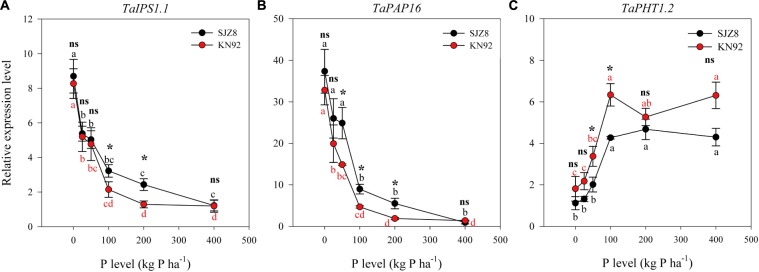
Relative expression levels of *TaIPS1.1* **(A)**, *TaPAP16* **(B)**, and *TaPHT1.2* **(C)** for SJZ8 (black) and KN92 (red) at anthesis stage in 2011. Values are means ± SE of four replicates. Within each cultivar, different lowercase letters indicate significant difference between P application levels (Turkey test, *P* < 0.05); ns and ^∗^ indicate no significant difference and significant difference, respectively, between SJZ8 and KN92 at the same P application level (Student *T*-test, *P* < 0.05).

Expression of all three tested genes in roots was significantly influenced by P application level, and except *TaIPS1.1*, expression of *TaPAP16* and *TaPHT1.2* was significantly influenced by cultivar (Table 5). Expression of *TaIPS1.1* in roots in response to the P application level was similar for the two cultivars, i.e., relative expression level was high when the P application level was 0 kg P ha^-1^ but decreased exponentially as the application level increased to 100 kg P ha^-1^ (Figure 4A). Expression of *TaIPS1.1* was higher for SJZ8 than KN92 at P application level of 100 and 200 kg P ha^-1^. Like *TaIPS1.1, TaPAP16* expression in roots of both cultivars was high when the P application level was 0 kg P ha^-1^ but decreased exponentially as the application level increased, and expression was higher for SJZ8 than KN92 when the P application level was ≤200 kg P ha^-1^ (Figure 4B). In contrast to *TaIPS1.1* and *TaPAP16*, expression levels of *TaPHT1.2* in roots of both cultivars gradually increased with increasing P application level up to 100 kg P ha^-1^ and then remained relatively stable, with higher values for KN92 than SJZ8 regardless of P application level (Figure 4C).

## Discussion

### The Two Wheat Cultivars Responded Differently to P Supply in Term of Root Morphology, Physiology, and Molecular Biology

P mobility in soil is very low, and most applied fertilizer P is generally found in the surface layer of the soil profile (Messiga et al., 2012). A plant’s ability to acquire soil P could be increased by alterations in root morphology and foraging (Lynch and Brown, 2001). As the P application level increased in the present study, RDW and RLD in the 0–60 cm soil layer for both wheat cultivars increased until a critical P application level (100 kg P ha^-1^) for optimal aboveground plant growth was obtained; higher P application levels did not further increase or even slightly reduced RDW and RLD. Although relatively more photosynthate was allocated to roots to increase the root/shoot ratio (*data not shown*) under a deficient P supply, the overall plant growth was depressed due to limited photosynthesis. Therefore, root growth was higher when the P supply was sufficient rather than deficient.

Arbuscular mycorrhizal fungi are widely known to colonize most plant roots and to develop wide networks of hyphae in the soil; by penetrating soil pores unavailable to the roots, AMF hyphae facilitate P acquisition by roots (Smith and Read, 2010). In contrast to root growth, AMF colonization of roots of both wheat cultivars was high with low P application levels and was inhibited by high P application levels, which is consistent with previous reports (Kahiluoto et al., 2001; Covacevich et al., 2007). When root growth without AMF colonization is sufficient for P uptake, reduced AMF colonization indicates an efficient utilization of carbon by plants, because AMF usually consume 4–20% of host plant photosynthate for their own growth (Bago and Shachar-Hill, 2000). Thus, increased root growth and reduced AMF colonization as influenced by changes in P application levels probably demonstrates an efficient strategy of root foraging for P acquisition with less carbon cost. Regarding cultivars, KN92 had better root growth and AMF colonization than SJZ8, particularly under a deficient P supply. This suggests that for the efficient acquisition of P, root morphological traits are more important to KN92 than SJZ8.

Synthesis and secretion of phosphatase by wheat roots has been reported to increase P availability to those roots (George et al., 2008). In the current study, APase activity in rhizosphere soil was increased by a low P application rate for both cultivars and especially for SJZ8, which was consistent with Ciereszko et al. (2011). Although an upregulation of the phosphatase encoding gene *TaPAP16* was observed for both cultivars when the P application rate decreased (Figure 4B), the increase in expression was more closely related to an increase in APase activity in the rhizosphere soil for cultivar SJZ8 than for KN92. This difference between the two wheat cultivars indicates a more important role of APase in the acclimation of cultivar SJZ8 to P deficiency.

Specific phosphate transporter proteins, including those in the PHT1 family, are required at the root/rhizosphere interface to take up P from soil (Poirier and Jung, 2015). Several genes encoding PHT1 transporters have been reported for wheat (Teng et al., 2017). In the current study, expression of *TaPHT1.2* in the roots of both cultivars was inhibited by P deficiency. Teng et al. (2017) recently reported that the expressions of *TaPHT1.2, TaPHT1.1/1.9*, and *TaPHT1.10* were root-specific and were lower when the P supply was low rather than high. These four genes are closely related to *HvPHT1.1* and *HvPHT1.2* in barley (Teng et al., 2017). In barley roots, AMF colonization can inhibit the responses of *HvPHT1.1* and *HvPHT1.2* to P deficiency (Glassop et al., 2005). Therefore, the inhibition of *TaPHT1.2* by P deficiency in the present study may have been partially due to increased AMF colonization under P deficiency. The high expression of *TaPHT1.2* and low AMF colonization under high P application rates indicated that the root-specific *TaPHT1.2* contributed to P acquisition under high P conditions. Furthermore, the higher expression of *TaPHT1.2* in KN92 than SJZ8 probably contributed to the higher PAE in KN92.

The previous paragraphs indicate that under P-deficient conditions, SJZ8 had better root physiological traits in terms of rhizosphere APase activity and *TaPAP16* expression, while KN92 had better root morphology and foraging as indicated by root growth and AMF colonization. This suggests that for P acquisition under P-deficient conditions, SJZ8 mainly depended on changes in root physiology, whereas KN92 mainly depended on changes in root morphology. Under high-P conditions, however, none of the measured root physiological or morphological traits differed between the two cultivars. After the vegetation stage, positive correlations were previously reported between P uptake and expression levels of *TaPHT1.2, TaPHT1.1/1.9*, and *TaPHT1.10* (Teng et al., 2017). High expression levels of *TaPHT1.2* under high-P conditions may contribute to efficient acquisition of P from soils for both cultivars, especially for cultivar KN92, which had higher expression of *TaPHT1.2* and higher P uptake.

### PAE and PUE in the Reproductive Stage Accounts for the Difference in P Efficiency Between the Two Wheat Cultivars

The responses of plant growth and the P-starvation marker gene *TaIPS1.1* to different P application levels demonstrate that SJZ8 and KN92 had similar yield potentials and reached optimal growth at the same critical P application level (around 100 kg P ha^-1^). At least from the anthesis stage on, however, PAE expressed as TPU in aboveground plant materials and plant PUE began to differ between SJZ8 and KN92. Although TPU was greater in KN92 than in SJZ8 (Table 2), the PUE of shoots and grain were higher in SJZ8 than in KN92, regardless of P application level (Table 3). These results support the view of Wang et al. (2010) concerning the importance of genotypic control in P efficiency.

The crop P requirement during post-anthesis growth is determined by the P required by the vegetative tissue to continue normal cellular function and by the P demand of the developing grain (Rose et al., 2007). Because the yield potentials were similar for SJZ8 and KN92 in the current study, P uptake and translocation after anthesis until maturity may partly explain the difference in PAE and PUE between the two cultivars in reproductive growth, which needs further research. In terms of economics, SJZ8 is superior to KN92 because it can produce a similar grain yield with less P uptake, reducing the removal of P from fields in both P-deficient and P-adequate scenarios. On low-P soils, however, the higher P levels in KN92 grain might benefit KN92 seedling vigor, a possibility that warrants further research.

It is noticed that plant growth, plant P concentration, total P uptake and P efficiency significantly differed between 2010 and 2011 season. Previous studies have shown that P nutrition could directly alter the pattern of tiller emergence and consequently influence the number of ears per unit of area, which plays an important role in wheat growth and yield potential (Rodríguez et al., 1999; Fioreze et al., 2012). Higher P levels could increase wheat tiller emergence, survival and yield (Fioreze et al., 2012). In the present study, both wheat cultivars had similar seedling density at sowing in each season (about 3.9 × 10^6^ seedling per hectare), but tillering of two cultivars differed between 2010 and 2011 seasons as affected by P application level. In 2010, with increasing P application, SJZ8 produced 6.6 × 10^6^–9.6 × 10^6^ tillers per hectare at vegetation and 3.8 × 10^6^–5.1 × 10^6^ ears per hectare at harvest, while KN92 produced 7.1 × 10^6^–1.1 × 10^7^ tillers per hectare at vegetation and 4.2 × 10^6^–5.5 × 10^6^ ears per hectare at harvest. In 2011, with increasing P application, SJZ8 produced 6.0 × 10^6^–1.5 × 10^7^ tillers per hectare at vegetation and 4.0 × 10^6^–7.8 × 10^6^ ears per hectare at harvest, while KN92 produced 7.0 × 10^6^–1.7 × 10^7^ tillers per hectare at vegetation and 4.2 × 10^6^–7.8 × 10^6^ ears per hectare at harvest (*data not shown*). Except plots with no P application, two wheat cultivars grown in plots under the same P fertilization level produced more tillers and ears in 2011 season than that in 2010 season, which probably indicates a part effect on plant growth of residual soil P from fertilization of previous season. As the present study only lasted two seasons, further study with longer time is needed to explore the influence of residual soil P from different P application levels on P efficiency and root characteristics.

## Conclusion

This study has provided field evidence of genotypic control of P efficiency for two wheat cultivars at all of the tested P supply levels. The study also indicated that the mechanisms underlying P efficiency differed between the two cultivars. PAE was higher in cultivar KN92 than in cultivar SJZ8, mainly because the PAE of KN92 benefitted from alterations in root morphology and increased AMF colonization under P-deficient conditions and from higher expression of the phosphate transporter gene *TaPHT1.2* under P-sufficient conditions. In contrast, PUE was higher in SJZ8 than in KN92, and the PAE of SJZ8 benefitted from root physiological adaptations to P deficiency (increased expression of the phosphatase encoding gene *TaPAP16* in roots and increased activity of APase in the rhizosphere). Because the current study only focused on root morphological, physiological, and molecular responses of the two wheat cultivars in early and middle growth stages, additional research on P uptake and translocation in the reproductive growth stage is needed to further clarify the genotypic control of P acquisition and utilization for both cultivars.

## Author Contributions

YD and X-PC designed the experiments. YD and WT carried out the experiments. YD and C-QZ analyzed the data and wrote the manuscript. Y-PT contributed to the interpretation of data and to the revision of the manuscript.

## Conflict of Interest Statement

The authors declare that the research was conducted in the absence of any commercial or financial relationships that could be construed as a potential conflict of interest.

## Abbreviations

AMF: arbuscular mycorrhizal fungi
APase: acid phosphatase
KN92: Kenong9204
PAE: phosphorus acquisition efficiency
PUE: phosphorus utilization efficiency
RDW: root dry weight
RLD: root length density
RPAE: phosphorus absorption efficiency of roots
SJZ8: Shijiazhuang8
TPU: aboveground total P uptake
